# Patients with severe low back pain exhibit a low level of physical activity before lumbar fusion surgery: a cross-sectional study

**DOI:** 10.1186/s12891-018-2274-5

**Published:** 2018-10-11

**Authors:** Hanna Lotzke, Max Jakobsson, Annelie Gutke, Maria Hagströmer, Helena Brisby, Olle Hägg, Rob Smeets, Mari Lundberg

**Affiliations:** 1 Institute of the Clinical Sciences, Department of Orthopaedics at Sahlgrenska Academy, University of Gothenburg, R-huset 7th Floor, SU/Mölndal, Göteborgsvägen 31, 431 80 Mölndal, Gothenburg, Sweden; 2 Spine Center Göteborg, Gruvgatan 8, 421 30 Västra Frölunda, Sweden; 3 Division of Home Medical Care, Department for Nursing and for the Care of the Elderly, Borås Stad, Borås, Sweden; 4 Division of Physiotherapy, Department of Health and Rehabilitation, Institute of Neuroscience and Physiology, University of Gothenburg, Box 455, 405 30 Gothenburg, Sweden; 5 Division of Physiotherapy, Department of Neurobiology, Care Sciences and Society, Karolinska Institutet, Alfred Nobels Alle 23, 141 83 Huddinge, Sweden; 6 Functional Area Occupational Therapy and Physiotherapy, Allied Health Professionals Function, Karolinska University Hospital, Stockholm, Sweden; 7 Department of Orthopaedics, Sahlgrenska University Hospital, Gothenburg, Sweden; 8 Department of Rehabilitation Medicine, Maastricht University, PO Box 616, 6200 MD Maastricht, The Netherlands; 9 CIR Revalidatie, Eindhoven, The Netherlands

**Keywords:** Accelerometer, Kinesiophobia, Steps per day, Chronic low back pain, Lumbar surgery

## Abstract

**Background:**

People with severe low back pain are at higher risk of poor health. Patients scheduled for lumbar fusion surgery are assumed to have low levels of physical activity, but few data exist. The aim of the study was firstly to investigate preoperative levels of objectively measured physical activity in patients with severe low back pain waiting for lumbar fusion surgery, and secondly to investigate whether factors in the fear-avoidance model were associated with these levels.

**Methods:**

We included 118 patients waiting for lumbar fusion surgery (63 women and 55 men; mean age 46 years). Physical activity expressed as steps per day and total time spent in at least moderate-intensity physical activity was assessed with ActiGraph GT3X+ accelerometers. The data were compared to the WHO recommendations on physical activity for health. Whether factors in the fear-avoidance model were associated with physical activity was evaluated by two different multiple linear regression models.

**Results:**

Ninety-six patients (83%) did not reach the WHO recommendations on physical activity for health, and 19 (16%) patients took fewer than 5000 steps per day, which indicates a sedentary lifestyle. On a group level, higher scores for fear of movement and disability were associated with lower numbers of steps per day.

**Conclusion:**

A high proportion of the patients did not reach the WHO recommendations on physical activity and are therefore at risk of poor health due to insufficient physical activity. We also found a negative association between both fear of movement and disability, and the number of steps per day. Action needs to be taken to motivate patients to be more physically active before surgery, to improve health postoperatively. There is a need for interventions aimed at increasing physical activity levels and reducing barriers to physical activity in the prehabilitation phase of this patient group.

**Trial registration:**

Current Controlled Trials ISCRTN 17115599, retrospectively Registered 18 may 2015.

## Background

Low back pain (LBP) causes more disability than any other condition, and the years lived with disability due to LBP lead to huge costs for society [1]. The number of patients who undergo elective spinal surgery for lumbar degenerative conditions is increasing worldwide [2]. Lumbar degenerative disease, including disc herniation, spinal stenosis and chronic low back pain due to degenerative disc disease, constitutes the major reason for elective lumbar spine surgery [3]. Despite increasingly better outcomes after surgery over the past years, 40% of patients still report dissatisfaction and experience problems with their function 1 year after spinal fusion surgery [4]. Patients with higher levels of disability before lumbar spine surgery are at a greater risk of poor surgical outcome [2, 5]. Moreover, self-reported poor health at the time of surgery has a negative impact on its outcome [2].

Physical activity has well-documented positive effects on health, it reduces premature mortality and should be recommended to all people regardless of condition [6–8]. People with LBP are at a higher risk of poor health (higher body mass index, higher blood pressure and a lower level of physical activity) due to this condition [9, 10]. In a recently published call for action on LBP, a stronger health focus was advocated [11]. The authors recommend that all actions in relation to LBP should be in synergy with the World Health Organization’s (WHO) action plans to prevent and control non-communicable diseases, such as having people reach the recommended level of physical activity. WHO recommends 150 min of at least moderate-intensity physical activity per week, performed in bouts of at least 10-min [12], equivalent to 7500 steps per day [13]. A prognostic longitudinal study, on patients seeking care for chronic LBP, showed that the group engaged in a higher activity level at baseline had less pain and disability at 12 months follow-up than the sedentary group [14]_._ Moreover, a meta-analysis concluded that patients with chronic LBP who also have high levels of disability are most likely to have a low physical activity level [15]. Patients with severe LBP waiting for lumbar fusion surgery are assumed to be physically inactive and deconditioned, and their physical activity status before surgery can be used as an indicator of health status, but there is a lack of data on the level of physical activity before surgery measured objectively.

There are some studies available that have studied physical activity in relation to lumbar spine surgery [16–20]. To our knowledge four studies have assessed the *preoperative* physical activity level [16, 18, 19, 21], in various lumbar degenerative conditions. None of these has investigated physical activity specifically for patients with degenerative disc disease. Since these patients differ from patients with spinal stenosis in that they are on average younger and have less leg pain [3], we hypothesised that the physical activity pattern would differ. Moreover, all but Norden et al. used self-reported data [18, 19, 21], which are considered less valid than those collected by objective measures such as an accelerometer [22]. We will therefore use accelerometers, considered to be one of the most valid measure, to accurately estimate levels of physical activity in daily life.

Various factors explain a low level of physical activity; the strongest correlates being age, sex, poor health status, low self-efficacy, and low motivation [23]. Moreover, pain itself is a barrier to being physically active. Factors included in the fear-avoidance model (e.g. catastrophizing, fear of movement and poor self-efficacy) can also be barriers to being physically active [24, 25]. Fear-avoidance beliefs have been found to be significant predictors of pain and functional outcomes for up to 2 years after lumbar spine surgery [26]. Until now, no studies have investigated the association between physical activity and fear-avoidance factors for patients with severe LBP scheduled for lumbar fusion surgery.

### Aim of the study

To investigate preoperative levels of objectively measured physical activity in patients with severe low back pain scheduled for lumbar fusion surgery, and further to investigate whether factors in the fear-avoidance model are associated with these levels.

## Methods

This cross-sectional observational study was conducted and reported according to the Strengthening the Report of Observational Studies in Epidemiology (STROBE Statement) [27]. The data in this study are baseline data collected in a randomised controlled trial before any treatment [28]*.*

### Patient selection

Included were patients scheduled for lumbar fusion surgery with severe LBP and degenerative changes of 1–3 segments of the lumbar spine. Patients with additional minor radiating symptoms will if necessary have a simultaneous surgical procedure for disc herniation, foraminal spinal stenosis or isthmic spondylolisthesis. Exclusion criteria were: previous decompression surgery for spinal stenosis; spinal malignancy; confirmed neurological or rheumatic disorder; deformities in the thoracolumbar spine; or poor understanding of the Swedish language.

### Procedure

One hundred and eighteen patients awaiting lumbar fusion surgery from two private spine clinics and a university hospital in Gothenburg, Sweden between April 1, 2014, and July 1, 2017, were included. At 8 to 12 weeks before surgery, the patients met an independent observer and received questionnaires and an accelerometer. The patients were instructed to wear the accelerometers for seven consecutive days during waking hours, and the device was attached to the patient’s non-dominant hip with an elastic band [29]. The device was to be removed before bathing, showering or swimming.

### Demographic data

Demographic data were collected by routine use of the Swedish Spine Register (Swespine) and included age, gender, self-reported weight and height (Body Mass Index (BMI), kg/m^2^), smoking status, educational level, sick leave, and previous surgery.

### Physical activity

Physical activity was measured by the triaxial accelerometer ActiGraph GT3X+ (Actigraph, Pensacola, FL). The accelerometer measures several physical activity domains, but for the purpose of this study, two were used. As a measure of total physical activity, steps per day was used. As a measure of intensity, minutes per week in at least moderate-intensity physical activity was used. Physical activity of at least moderate-intensity can be compared to a brisk walk [13].

### Fear-avoidance factors

**Fig. 1:**
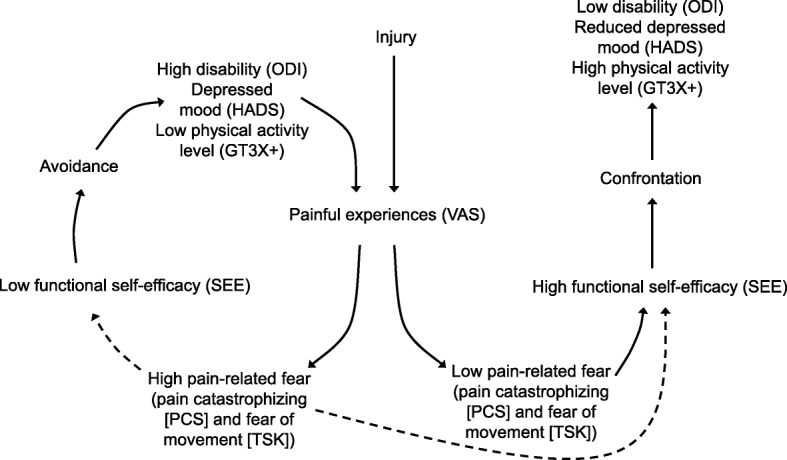
Revised fear-avoidance model. VAS, Visual Analogue Scale; PCS, Pain Catastrophizing Scale; TSK, Tampa Scale for Kinesiophobia; SEES, Self-Efficacy for Exercise Scale; ODI, Oswestry Disability Index; HADS, Hospital Anxiety and Depression Scale; GT3X+, physical activity of least moderate-intensity, steps per day

Data on fear-avoidance factors were collected by patient-reported outcome measures (PROMs). The choice of PROMs was based on the revised fear-avoidance model presented in Lotzke et al. [28] as displayed in Fig. 1.

Back and leg pain intensity levels over the last week were measured using 100-mm Visual Analogue Scales (VAS). An intensity score of less than 10 mm was taken as no pain and 20–25 mm or above was taken as relevant pain [30]. Pain catastrophizing was measured using the Pain Catastrophizing Scale (PCS). Total scores range from 0 to 52, with 0 indicating no catastrophizing. A cut-off score of 20 points correspond to a moderate level of catastrophizing [31]. Fear of movement was rated using the Tampa Scale of Kinesiophobia (TSK). Total scores ranges from 17 to 68, higher scores indicating higher level of kinesiophobia. A cut-off score of < 37 points was used to indicate kinesiophobia [32]. Self-efficacy related to exercise was measured using the Self-Efficacy for Exercise Scale (SEES). Total scores range from 0 to 90, higher scores indicating higher levels of self-efficacy [33]. Anxiety and depression were assessed using the Hospital Anxiety and Depression Scale (HADS). Total scores range from 0 to 21, higher scores indicating a higher level of anxiety or depression [34]. Disability was measured using Version 2.0 of the Oswestry Disability Index (ODI). Values on the ODI of 21- to 40 represent moderate disability; 41–60, severe disability; 61–80 incapacitating disability; and 81–100 restricted to bed [35]. Health-related quality of life was measured using the European Quality of Life 5 Dimensions Questionnaire (EQ5D index). Possible scores range from − 0.59 to 1.0, where 1.0 represents optimal health [36]. Details of the psychometric properties of the questionnaires are reported elsewhere [28].

### Statistical analysis

Data analysis was performed using the statistics software SPSS 24 (IBM Corporation, New York, NY). Descriptive statistics for all variables are presented as frequency (proportion) or mean (SD) depending on the data level and data distribution. The number of internal missing data is shown in the tables separately.

The raw data from the accelerometer were downloaded and checked for wear time with the Actilife v6.13.0 software. A valid data set for a patient was defined as wear time of at least 10 h per day for a minimum of 4 days [29, 37]. Data were analysed on a minute-by-minute basis in Actilife to generate the total physical activity (steps per day) and physical activity intensity (minutes per week in at least moderate-intensity physical activity). The threshold for moderate-intensity physical activity was set at 2020 counts per minute on the vertical axis, as recommended by Troiano et al. [38]. The physical activity intensity was presented both as total accumulated minutes per week and as minutes per week accumulated in at least 10-min bouts. A “10-min bout” was defined as a 10-min period with an interruption of no more than 2 min below the threshold of 2020 counts per minute [38]. We then calculated the proportions of patients who reached the WHO physical activity recommendations in 10-min bouts. Further, we calculated the proportions of patients that reached ≥ 7500 steps per day (physically active lifestyle), 5000–7499 steps per day (low active lifestyle) and < 5000 steps per day (sedentary lifestyle) [12, 13, 39].

The association between the fear-avoidance factors and the physical activity variables were investigated in two multiple linear regression models: total physical activity, and physical activity intensity in 10-min bouts were used as the dependent variables. A purposeful selection method was used to select independent variables for the final multiple regression models [40]. The maximum number of potential independent variables was calculated based on power level = 0.8, alpha = 0.05, and anticipated effect size = 0.15 (“moderate”) [41]. This calculation yielded a maximum of ten potential independent variables to be included in the regression analyses. First, independent variables associated with the dependent variable at a *p*-value > 0.25 in univariate regression analyses were excluded from the subsequent step of the analysis. Independent variables were excluded in this fashion regardless of whether they were fear-avoidance factors or “potential” confounders (age, gender, BMI). Second, the remaining independent variables were included in a backward multiple regression analysis in which independent variables with a *p*-value > 0.15 were removed, provided that the beta coefficient of the remaining independent variables did not change by more than 15%. Third, the independent variables excluded in the initial univariate regression analyses were added back to the multiple regression model one by one, only being kept if they had a *p*-value ≤ 0.15. This final step was performed to identify independent variables that were potentially significant in the presence of the other independent variables but not to the dependent variable alone [40]. Any remaining confounders (age, gender, or BMI) in the final model were not interpreted in the results section as they were only added to adjust the model. The independent variables in the final model were controlled for multicollinearity and the standardized residuals from the regression model were checked for normality and heteroscedasticity.

**Fig. 2:**
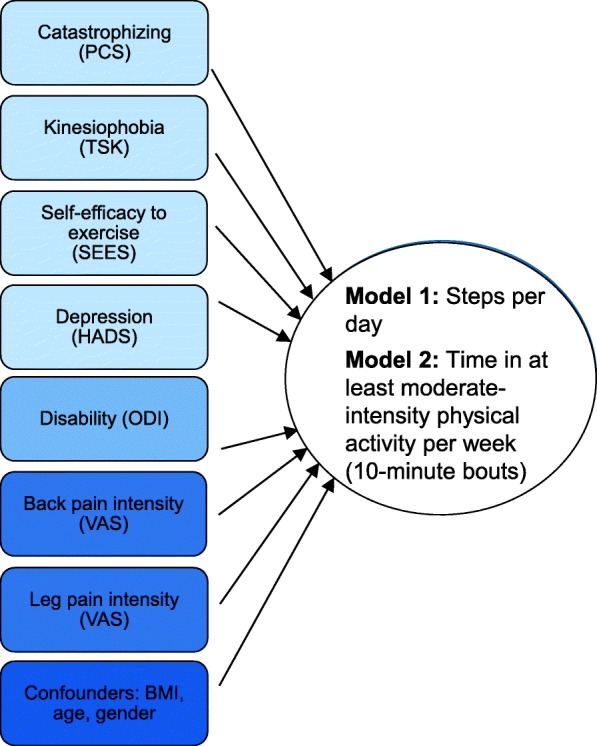
Overview of the variables in the regression models. PCS, Pain Catastrophizing Scale; TSK, Tampa Scale for Kinesiophobia; SEES, Self-Efficacy for Exercise Scale; HADS, Hospital Anxiety and Depression Scale; ODI, Oswestry Disability Index; VAS, Visual Analogue Scale

The standardised residuals in the multiple linear regression analysis of total physical activity (steps per day) and physical activity intensity in 10-min bouts were not normally distributed, and the variables were therefore transformed into their natural logarithms. The standardised residuals of the physical activity intensity in 10-min bouts were still not normally distributed after the transformation and the variable was therefore not further investigated (Fig. 2).

## Results

The study comprised 63 women and 55 men with a mean age of 46 (SD = 8) years. Forty-one (35%) patients were on sick leave, and 11 (9%) patients had had previous lumbar spine surgery (Table 1).

**Table 1:** Socio-demographics and health characteristics for all patients

	Sample *n* = 118	Women *n* = 63	Men *n* = 55
Age, mean (SD)	45.7 (8.3)	45.0 (8.6)	46.5 (8.1)
Body Mass Index, mean (SD)	26.3 (3.7)	25.5 (4.0)	27.3 (3.1)
Smoking^b^, frequency (%)	8 (6.8%)	4 (6.3%)	4 (7.4%)
Education^b^, frequency (%)			
Elementary school	7 (6.0%)	2 (3.2%)	5 (9.1%)
High school	51 (43.6%)	26 (41.3%)	25 (45.4%)
University	42 (35.9%)	28 (44.4%)	14 (25.9%)
Vocational education	17 (14.5%)	7 (11.1%)	10 (18.2%)
Sick leave^a^, frequency (%)			
No sick leave	75 (64.7%)	35 (56.5%)	40 (74.1%)
Full-time	23 (19.8%)	14 (22.6%)	9 (16.7%)
Part-time	16 (13.8%)	11 (17.7%)	5 (9.3%)
Other disease	2 (1.7%)	2 (3.2%)	0 (0.0%)
Previous lumbar disc surgery for radiculopathy, frequency (%)	11 (9.3%)	7 (11.1%)	4 (7.3%)

^a^*n* = 116

^b^*n* = 117; values correspond to mean (SD) or frequency (percent)

Eighty-seven (74%) patients had suffered from back pain for more than 2 years. On average, the patients had a moderate disability (ODI), moderate level of back pain (VAS), high fear of movement (TSK), moderate pain catastrophizing thoughts (PCS), and reduced health-related quality of life (EQ5D index) (Table 2).

**Table 2:** Values for accelerometer data and fear-avoidance factors for all patients

	All *n* = 118	Women *n* = 63	Men *n* = 55
Accelerometer data^a^			
Time spent in MVPA per week (10-min bouts)	81.7 (116.9)	87.4 (107.9)	75.1 (127.1)
Time spent in MVPA per week (total accumulated)	197.6 (141.3)	187.6 (134.1)	209.1 (149.7)
Steps/day	7493.5 (2645.4)	7553.6 (2728.0)	7424.4 (2571.0)
Pain intensity – back (VAS)	61.1 (19.4)	63.3 (17.7)	58.7 (21.0)
Pain intensity – leg (VAS)^b^	35.4 (29.7)	36.0 (28.8)	34.7 (30.9)
Disability (ODI)	37.8 (12.4)	38.2 (11.7)	35.3 (13.0)
Pain catastrophizing (PCS)	22.8 (8.1)	22.7 (8.1)	22.9 (8.2)
Fear of movement (TSK)	38.1 (8.4)	35.9 (7.7)	40.6 (8.6)
Self-efficacy for exercise (SEES)	61.2 (20.5)	62.6 (20.1)	59.5 (20.9)
Depression (HADS)	5.4 (3.6)	5.4 (2.9)	5.4 (4.3)
Anxiety (HADS)	6.6 (3.7)	6.5 (3.7)	6.7 (3.7)
Health related quality of life (EQ5D index)^b^	0.49 (0.29)	0.51 (0.28)	0.47 (0.30)

Values correspond to mean (SD)

*MVPA* physical activity of least moderate-intensity, *VAS* Visual Analogue Scale, *ODI* Oswestry Disability Index, *PCS* Pain Catastrophizing Scale, *TSK* Tampa Scale for Kinesiophobia, *SEES* Self-Efficacy for Exercise Scale, *HADS* Hospital Anxiety and Depression Scale, *EQ5D index* Health Related Quality of Life

^a^*n* = 116

^b^*n* = 117

### Physical activity results

Out of the 118 patients, 116 gave valid data as measured by the accelerometer.

#### Physical activity intensity in relation to WHO health recommendations

**Fig. 3:**
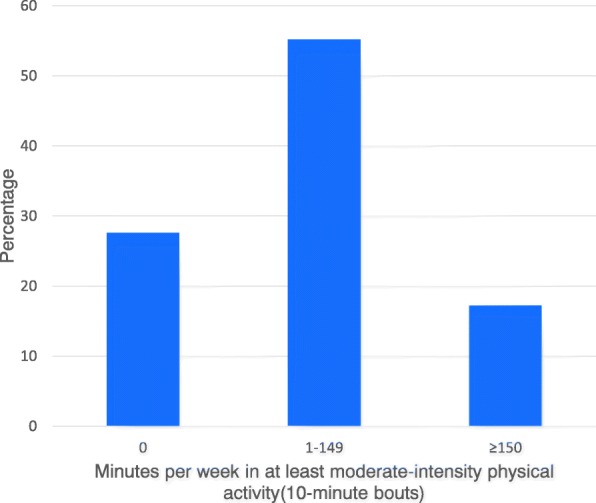
Histogram of at least moderate- intensity physical activity per week (10-min bouts)

In terms of physical activity intensity (10-min bouts), only 20 patients (17%) reached the WHO recommendations on physical activity for health (≥ 150 min per week). Of the 96 patients who did not reach the recommendations, 32 patients (28%) spent zero minutes per week in at least moderate-intensity physical activity (10-min bouts) and 64 patients (55%) spent between 1 and 149 min per week in this (Fig. 3).

#### Total physical activity

**Fig. 4:**
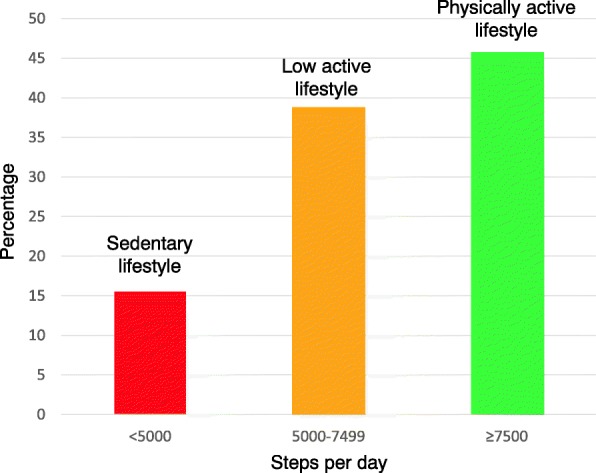
Histogram of steps per day

In terms of total physical activity, 19 patients (16%) took less than 5000 steps per day (sedentary lifestyle), 44 patients (38%) between 5000 and 7499 steps per day (low active lifestyle), and 53 (46%) patients walked ≥ 7500 steps (physically active lifestyle) (Fig. 4) [13, 42].

### Associations between factors in the fear-avoidance model and steps per day (dependent)

The final model contained back pain, fear of movement, disability, and BMI (Table 3). Out of these, fear of movement and disability level was found to be significantly associated with the dependent log-transformed variable steps per day*.* At group level, a 10 points lower level of fear of movement (TSK) was associated with an 8.6% greater number of steps per day, as was a 10 points lower level of disability (ODI).

**Table 3:** Multiple linear regression analysis results with the dependent variable “steps per day” (log-transformed)

Variables	Unstandardized Beta	Standardized Beta	P	95% CI for B	
				Lower	Upper
(Constant)	9.930		< 0.001	9.390	10.470
BMI	−0.021	−0.203	0.020	−0.038	−0.003
ODI	−0.009	−0.295	0.006	−0.015	−0.003
VASBack	0.002	0.109	0.293	−0.002	0.006
TSK	−0.009	−0.197	0.034	−0.017	−0.001

*BMI* body mass index, *ODI* Oswestry Disability Index, *VASBack* Visual Analogue Scale – back pain, *TSK* Tampa Scale for Kinesiophobia

## Discussion

This study demonstrates that the majority of the patients, scheduled for lumbar fusion surgery, were insufficiently active, according to the variable of “physical activity intensity” (10-min bouts). Further, more than half of the patients did not reach the level of a physically-active lifestyle measured by the variable “steps per day”. Moreover, their steps per day was negatively associated with both fear of movement and disability.

These are some of the first results in the world, based on objective measures, investigating the physical activity status in patients with severe LBP scheduled for lumbar fusion surgery. As many as 83% did not reach the WHO recommendations of physical activity for health. As compared to a “healthy population” in Sweden [43] and Germany [44] this suggests a large proportion of the patients in our study has therefore an increased risk of developing additional disease and poor health [7]. This suggests that a majority of the patients in our study do not reap the health benefits of being sufficiently physically active and are therefore at risk of developing additional disease and poor health [7]. Similar findings have been found by Mobbs et al., [16] and Norden et al., [21]. Mobbs et al. studied a mixed group of patients with degenerative lumbar disorders and found that the mean value for the variable “steps per day” (5255 steps) was below the threshold for a physically active lifestyle [16]. They also used an accelerometer but only presented the variable as “steps per day”. Norden et al. showed that only 4% of their patients with spinal stenosis met the physical activity recommendations before decompression surgery [21]. Moreover, a recently published systematic review and meta-analysis by Koenders et al., [45], concluded that *“research should provide more information regarding the clinical care pathway, psychosocial and physical conditions of the patients undergoing first time lumbar fusion surgery, and without this information, it remains impossible to improve lumbar fusion surgery management”*. We would argue that the new knowledge presented in our manuscript will help clinicians in structuring the rehabilitation program including the prehabilitation phase. Our results indicate that the physical activity level was low for a high proportion of our study group. If these patients remain inactive after surgery, maintaining their preoperative low activity levels and sedentary lifestyles, they will increase their risk of developing additional disease. By accurately measuring the physical activity level before surgery, the patient’s physical activity behaviour might be addressed preoperatively, increasing the chance of a healthier outcome of surgery.

There is a dose-response relationship between physical activity and health, i.e., the more physical activity, the better the health improvements. The largest health effect is gained by encouraging the most physically-inactive persons to become more physically active [46]. A high proportion of our study population was physically active at a lower level, and 19 patients (16%) were identified as having a sedentary life style, using the variable “steps per day”. The largest health effects are potentially to be gained by encouraging this subgroup to be more physically active. There was a large variation in physical activity levels in our patient group. Twenty patients (17%) were highly physically active and reached the WHO recommendations, and nearly half of the study group (46%) reached the physically-active lifestyle, as measured by the variable “steps per day”. “Steps per day” is not part of the WHO recommendations but offers a new way of translating the WHO guidelines in to a measurement that is easy to understand and communicate to the patient [13]. This measurement of physical activity is a valuable tool when designing personalised physical activity goals for patients that need or want to improve their physical activity levels.

The results in the present study showed a negative association between the amount of steps per day and both fear of movement and disability. This means that, if the levels of fear of movement and disability decrease, the number of steps per day might increase. Similar results were seen in a study by Donnarumma et al. of patients awaiting lumbar decompression and fusion surgery [47]. Their results demonstrated a significant correlation between self-reported low levels of physical activity and higher levels of disability, as well as higher levels of fear of movement [47]. Physical activity is, however, a complex behaviour and increasing physical activity is part of a behaviour change process. We also found that our study group presented with fear of movement (kinesiophobia), catastrophizing thoughts and reduced self-efficacy for exercise. Factors such as fear of movement and low self-efficacy are identified as barriers to physical activity. It has been suggested elsewhere that targeting fear of movement before surgery will increase the likelihood of a better functional outcome [48]. Since the largest health benefits are potentially gained by people with a less active or sedentary lifestyle, it is important to identify such patients, as well as those with fear-avoidance behaviours in the prehabilitation phase. A person-centred approach in the prehabilitation phase could help guide clinicians in structuring a personalised prehabilitation plan, focusing on each patient’s needs and preferences. Such an intervention program has been set up by the authors [28], and its results are under investigation.

### Strengths and limitations of the study

The major strength of the present study is that we measured the physical activity level with an accelerometer *before* surgery. In terms of external validity, some aspects need to be addressed. The study population is a well-defined group with strict inclusion and exclusion criteria. This group, with degenerative disc disease, is comparable to other patients (age, gender, pain duration of back/leg, and pain intensity back) with the same diagnosis presented included in The Swedish Spine Register (Swespine). Our sample is somewhat healthier (higher level of EQ5D, lower ODI score, lower score of leg pain, and lower number of previously-performed back surgeries), compared with the normative data in Swespine. The most likely reason is that these patients have agreed to participate in an active prehabilitation program, and, as has been shown in previous studies, people who are more active are also more likely to participate in such an intervention [49]. One therefore need to consider these differences when interpreting our data.

Another strength is the use of accelerometers, considered to be one of the most valid way to measure physical activity accurately [22]. In contrast to questionnaires, accelerometers are not subject to recall and response bias, and may, therefore, provide a more accurate estimate of physical activity [50]. Nevertheless, though they measure physical activity in a more objective way, accelerometers worn on the hip mainly measure ambulatory activities and cannot fully capture activities that cause little movement of the body’s center of gravity such as cycling. Moreover, as the accelerometer had to be removed in water, activities such as swimming cannot be measured. For some individuals, accelerometer measurements may therefore lead to an underestimation of physical activity, but no data were collected to assess this [51]. Another strength is the investigation of the factors in the fear-avoidance model and the new knowledge that scores on these outcome measures bring in this population. Patients scheduled for lumbar fusion surgery have a low level of physical activity, reduced self-efficacy for exercise as well as high levels of kinesiophobia and catastrophizing thoughts. This information will give a more extensive description of the population of interest and the risk factors associated with this subgroup of degenerative lumbar disorders. Since this is a cross-sectional study, we cannot draw any conclusions of causality which can be seen as a limitation of the study.

## Conclusions

The current study shows that a high proportion of the patients do not match the WHO health recommendations on physical activity and are therefore at risk of poor health due to insufficient physical activity. Moreover, we also found a negative association between both fear of movement and disability, and the amount of steps per day. Actions need to be taken to motivate patients to be more physically active before surgery. To successfully address low physical activity in patients with severe LBP awaiting lumbar fusion surgery, a better understanding of the reasons behind this low level may be important as an aid to increase the chance of a healthier outcome following surgery. There is a need for interventions aiming at increasing physical activity and to reduce barriers to physical activity in the prehabilitation phase for this patient group.

## Abbreviations

BMI: Body mass index
EQ5D index: Health related quality of life
HADS: Hospital Anxiety and Depression Scale
LBP: Low Back Pain
ODI: Oswestry Disability Index 2.0
PCS: Pain Catastrophizing Scale
PROMs: Patient- reported outcome measures
SEES: Self-Efficacy for Exercise Scale
Swespine: Swedish Spine Register
TSK: Tampa Scale for Kinesiophobia
VAS: Visual Analogue Scale
WHO: World Health Organization
